# Acupuncture effects on emotional reactivity of youth with self-reported depressive symptoms

**DOI:** 10.1093/psyrad/kkaf006

**Published:** 2025-04-04

**Authors:** Qian Wu, Dehui Lin, Xiuyun Wen, Shanze Wang, Derek Fisher, Wenbin Fu, Jiajin Yuan

**Affiliations:** Department of Acupuncture and Moxibustion, The Second Affiliated hospital of Guangzhou University of Chinese Medicine (Guangdong Provincial Hospital of Chinese Medicine), Guangzhou 510030, China; Department of Acupuncture and Moxibustion, The Second Affiliated hospital of Guangzhou University of Chinese Medicine (Guangdong Provincial Hospital of Chinese Medicine), Guangzhou 510030, China; School of Health Science, Guangdong Pharmaceutical University, Guangzhou 51006, China; Department of Acupuncture and Moxibustion, The Second Affiliated hospital of Guangzhou University of Chinese Medicine (Guangdong Provincial Hospital of Chinese Medicine), Guangzhou 510030, China; Department of Psychology, Mount Saint Vincent University, Halifax, Nova Scotia B3M 2J6, Canada; Department of Acupuncture and Moxibustion, The Second Affiliated hospital of Guangzhou University of Chinese Medicine (Guangdong Provincial Hospital of Chinese Medicine), Guangzhou 510030, China; Innovative Research Team of Acupuncture for Depression and Related Disorders, The Second Affiliated Hospital of Guangzhou University of Chinese Medicine (Guangdong Provincial Hospital of Chinese Medicine), Guangzhou 510030, China; Sichuan Key Laboratory of Psychology and Behavior of Discipline Inspection and Supervision, Institute of Brain and Psychological Sciences, Sichuan Normal University, Chengdu 610066, China

**Keywords:** depression, emotional reactivity, ERP, oddball, non-specific effect

## Abstract

**Background:**

Emotional symptomatology is a hallmark of depression. Antidepressant often fail to effectively target emotional blunting, while acupuncture, by contrast, has emerged as a promising alternative. However, the exact electrophysiologic mechanisms remain unclear. This study aimed to investigate how acupuncture influences emotional reactivity in youth with self-reported depressive symptoms.

**Methods:**

A modified oddball paradigm incorporating a negative emotional valence deviant, combined with event-related potential analysis, was used to measure emotional reactivity before and after intervention. Seventy individuals exhibiting depressive symptoms in the previous 2 weeks, were randomly assigned to either a verum or sham acupuncture group. Electroencephalogram data from 59 participants were analyzed following preprocessing and quality assessment. Occipital P1, N170, frontal N1, N2, and parietal P3 components were extracted. The Positive and Negative Affect Schedule (PANAS) was completed after each oddball session. The Massachusetts General Hospital Acupuncture Sensation Scale (MASS) was completed after each intervention session.

**Results:**

The MASS Index was significantly higher in the verum group. However, significant increases in occipital P1, N170, frontal N1, N2, and parietal P3 amplitudes for high-negative, mild-negative, and neutral pictures were observed after the intervention in both the verum and sham groups, with no significant difference between the groups. Additionally, both groups induced PANAS changes, and positive effect changes were significantly correlated with N170 and P1 (in response to high-negative pictures) changes in the sham group.

**Conclusion:**

Acupuncture altered emotional reactivity in youth with depressive symptoms, highlighting its potential role, albeit possibly non-specific, in depression prevention and treatment.

## Introduction

Depression, a major global mental health issue, affects over 300 million people worldwide, characterized by persistent mood disturbances, decreased interest, and anhedonia, with the potential for progression to suicidal thoughts in severe cases (Friedrich, 2017; World Health Organization, 2017). Emotional dysfunction is a key feature of depression, with the relationship between emotionality and major depressive disorder (MDD) persisting even after accounting for initial depression severity (Morris *et al*., 2009). A growing body of research suggests that depression is associated with a general reduction in response to positive and negative emotional stimuli, exhibiting a pattern known as emotion context insensitivity (Bylsma *et al*., 2021; Rottenberg *et al*., 2005; Rottenberg, 2017). Notably, depression impairs emotional reactivity, even in subthreshold states (Li *et al*., 2023; Moran *et al*., 2012). This pattern of reduced emotional reactivity, often termed emotional blunting, serves both as a symptom and as a risk factor (Bylsma *et al*., 2021; Dell'Acqua *et al*., 2023), playing a key role in the development of depressive disorders.

Pharmacotherapy, primarily with selective serotonin reuptake inhibitors (SSRIs), is the main treatment for depression (Brown *et al*., 2019; Flint and Banerjee, 2024; Hetrick *et al*., 2021; Kruizinga *et al*., 2021). However, it has limitations, including low response rates, delayed therapeutic effects, and adverse side effects (Arroll *et al*., 2005; Rush *et al*., 2003). Notably, around half of patients with depression report emotional blunting as a side effect of antidepressants (Goodwin *et al*., 2017). Even with significant symptom reduction, studies using the startle modulation paradigm show that pharmacotherapy does not normalize emotional responses in comparison to healthy controls (Dichter *et al*., 2004). While emotional numbing is recognized in depression, evidence suggests it can also result from monoaminergic antidepressants (Goodwin *et al*., 2017). Pronounced emotional blunting is associated with less satisfactory symptom remission, a more negative perception of one's condition, and potential treatment discontinuation (Goodwin *et al*., 2017). Other Western approaches, such as psychotherapy, offer significant benefits by facilitating emotional processing without the side effects associated with medication (Cuijpers*et al*., 2020), although this typically requires substantial time and patient engagement. Physical therapies also show promise; treatments such as transcranial magnetic stimulation have demonstrated efficacy (Miron *et al*., 2021; Xiao *et al*., 2024), yet some patients experience mild adverse effects including headache. Exercise interventions can effectively reduce depressive symptoms (Correia *et al*., 2024), but patient motivation often presents a significant barrier. Given the diverse benefits and challenges associated with existing therapies, there remains an ongoing need to explore alternative therapeutic options.

In this context, acupuncture has emerged as a promising complementary approach, with growing evidence supporting its benefits in addressing depression (Armour *et al*., 2019; Li *et al*., 2023; Qaseem *et al*., 2023; Ravindran *et al*., 2016; Smith *et al*., 2018), prompting calls for further research into its therapeutic advantages (NICE, 2022). Neuroimaging studies suggest that acupuncture's antidepressant effects may be mediated by the frontal lobe and limbic system (Fang *et al*., 2009; Wong *et al*., 2023; Zhang J *et al*., 2021), areas critical for emotional processing (Joseph, 1992; Ledoux, 1998). There are also reports of single-session acupuncture producing immediate effects on the left dorsolateral prefrontal cortex (left-DLPFC) in patients with major depressive disorder (Zhang T *et al*., 2021), indicating acupuncture's ability to influence emotional functions. However, exploration of acupuncture's impact on emotional reactivity in depression remains in its early stages.

The primary aim of this study was to investigate the effect of acupuncture on emotional reactivity in young individuals with self-reported depressive symptoms. To this end, we employed a visual oddball task with concurrent electroencephalogram (EEG) recording. This paradigm, simulating natural emotional occurrences within a non-emotional cognitive context (Delplanque *et al*., 2005; Yuan *et al*., 2007), reliably elicits attention-related N2 and cognition-related P3 components sensitive to emotional variability (Lou *et al*., 2016; Yuan *et al*., 2009, 2012, 2021). Notably, previous research has observed that the P300 component decreases in bimodal oddball tasks among individuals with MDD (Nan *et al*., 2018). Moreover, N1 decreases in response to emotional pictures in adolescents with depressive emotional tendencies (Wang Q *et al*., 2022), and thus we also analyzed early components such as P1, N1, and N170. We hypothesized that depressed individuals exhibit reduced sensitivity to naturally occurring negative emotional stimuli. By assessing pre- and post-acupuncture changes, with sham acupuncture as a control, we aimed to explore the therapeutic potential of acupuncture in modulating emotional reactivity in depression.

## Materials and methods

### Participants

The study was approved by the Ethics Committee of the Guangdong Provincial Hospital of Traditional Chinese Medicine, China (ZF2023-026-01). A cohort of 70 individuals with depressive symptoms in the past 2 weeks, but without a clinical diagnosis of depression, were recruited both online and offline. Depressive symptoms were assessed using the Center for Epidemiologic Studies Depression Scale (CES-D) (Radloff, 1977), and participants with a score of 16 or higher were included. Given the frequent co-occurrence of depression, anxiety, and insomnia (Freeman *et al*., 2020; Pearson *et al*., 2006; Yang *et al*., 2023), and the overlapping neural mechanisms between sleep wakefulness and stress-induced wakefulness (Ren *et al*., 2018; Zhao *et al*., 2022), scores from the Generalized Anxiety Disorder-7 scale (GAD-7) (Kroenke *et al*., 2007) and Pittsburgh Sleep Quality Index (PSQI) (Buysse *et al*., 1989) were collected for comprehensive assessment. Eleven participants were excluded due to discomfort during the experiment (two participants), narcolepsy (one participant), and poor data quality (eight participants, identified online and offline). The remaining 59 participants (29 in the verum group and 30 in the sham group), aged 20–31 years, were included in the analysis. The demographics of the two groups are shown in Table 1.

**Table 1:** Demographics.

t-test	Verum (*n* = 29)	Sham (*n* = 30)	Difference	*P*
Age (years)	25.1 (24.1, 26.1)	23.6 (22.5, 24.7)	−1.5 (−3.0, −0.0)	0.044*
CES-D	21.5 (20.1, 22.9)	22.9 (21.4, 24.4)	1.4 (−0.7, 3.5)	0.189
GAD-7	5.8 (4.4, 7.2)	6.0 (4.4, 7.6)	0.2 (−2.0, 2.4)	0.850
PSQI	14.7 (11.9, 17.5)	15.7 (13.7, 17.7)	1.0 (−2.4, 4.5)	0.560
**Fisher's exact test**			**Odds ratio**	***P***
Male	10	10	1.0 (0.3, 3.5)	1.000
Acu Naïve	10	15	0.5 (0.2, 1.7)	0.295
Married	2	1	2.1 (0.1, 130.9)	0.612
**Mann–Whitney U test**			**U statistic**	***P***
Education			409	0.644
– Postgraduate	3	4		
– Undergraduate	19	20		
– Below	7	6		

Note: Measurement are data described by mean (95% confidence interval); count data are described by counts.

### Procedure

**Figure 1:**
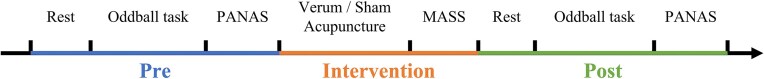
Study procedure.

Upon arrival, participants provided informed consent. As shown in Fig. 1, an intervention session was conducted between two task sessions. Each task session began with a 5-min rest period with eyes closed, followed by the oddball task. The Chinese version of the Positive and Negative Affect Schedule (PANAS) (Huang *et al*., 2003; Watson *et al*., 1988) was used to measure immediate emotions at the end of each task session. During the intervention session, participants received either verum or sham acupuncture. The Chinese version of the Massachusetts General Hospital Acupuncture Sensation Scale (MASS) (Kong *et al*., 2007; Yu *et al*., 2012) was completed at the end of the intervention session, and the MASS Index was calculated using all 12 terms of the scale. The study employed a 2 × 2 × 3 mixed design, with intervention type (verum vs. sham acupuncture) as a between-subject factor, and task session (pre-intervention vs. post-intervention) and emotional intensity (high-negative, mild-negative, and neutral) as within-subject factors.

### Oddball task

**Figure 2:**
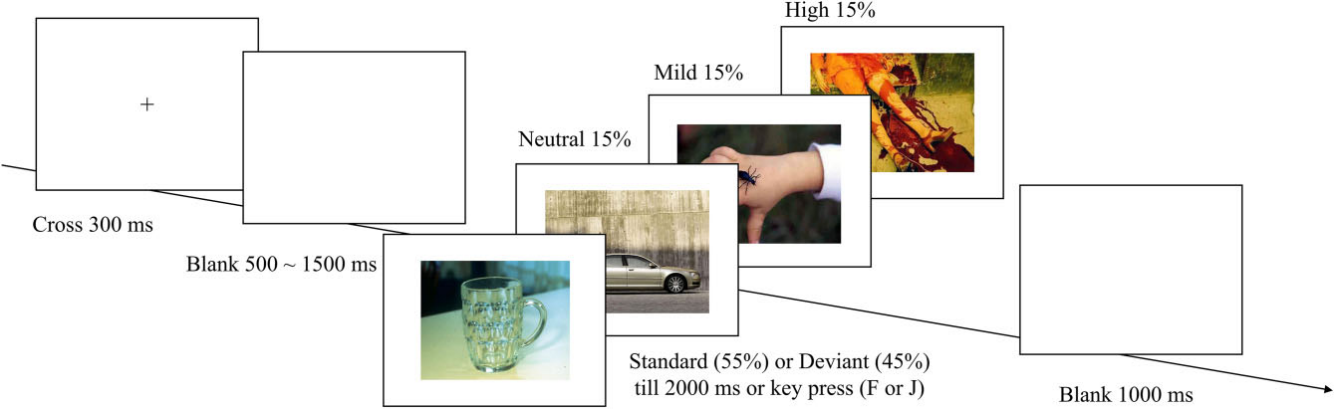
Trial structure and stimulus samples.

A modified oddball task was conducted in accordance with established protocols (Delplanque *et al*., 2005; Yuan *et al*., 2007). The task comprised four blocks, each containing 100 trials (55 standard and 45 deviant pictures). A consistent natural scene of a cup served as the standard stimulus throughout the experiment (Fig. 2). Deviant stimuli consisted of 90 pictures sourced from the Chinese Affective Picture System (Bai *et al*., 2005), categorized by emotional intensity into three groups: 30 high-negative, 30 mild-negative, and 30 neutral pictures. Each block presented 15 pictures per emotional intensity category. Blocks 3 and 4 repeated the full set of deviant pictures from earlier blocks. As shown in Fig. 2, each trial began with a 300-ms fixation cross, followed by a blank screen lasting randomly between 500 and 1500 ms. Subsequently, the picture was presented for 2000 ms. Participants were instructed to respond as rapidly and accurately as possible: the “F” key with their left index finger for standard stimuli and the “J” key with their right index finger for deviant stimuli. Stimulus presentation order was fully randomized per participant. The standard picture in the practice was the same as that in the formal experiment while deviant stimuli were neutral pictures that were not presented in the subsequent experiment. The picture was assessed for its valence and arousal on a nine-point scale with a large sample of Chinese participants in a previous survey (Long *et al*., 2015).

### Intervention

**Figure 3:**
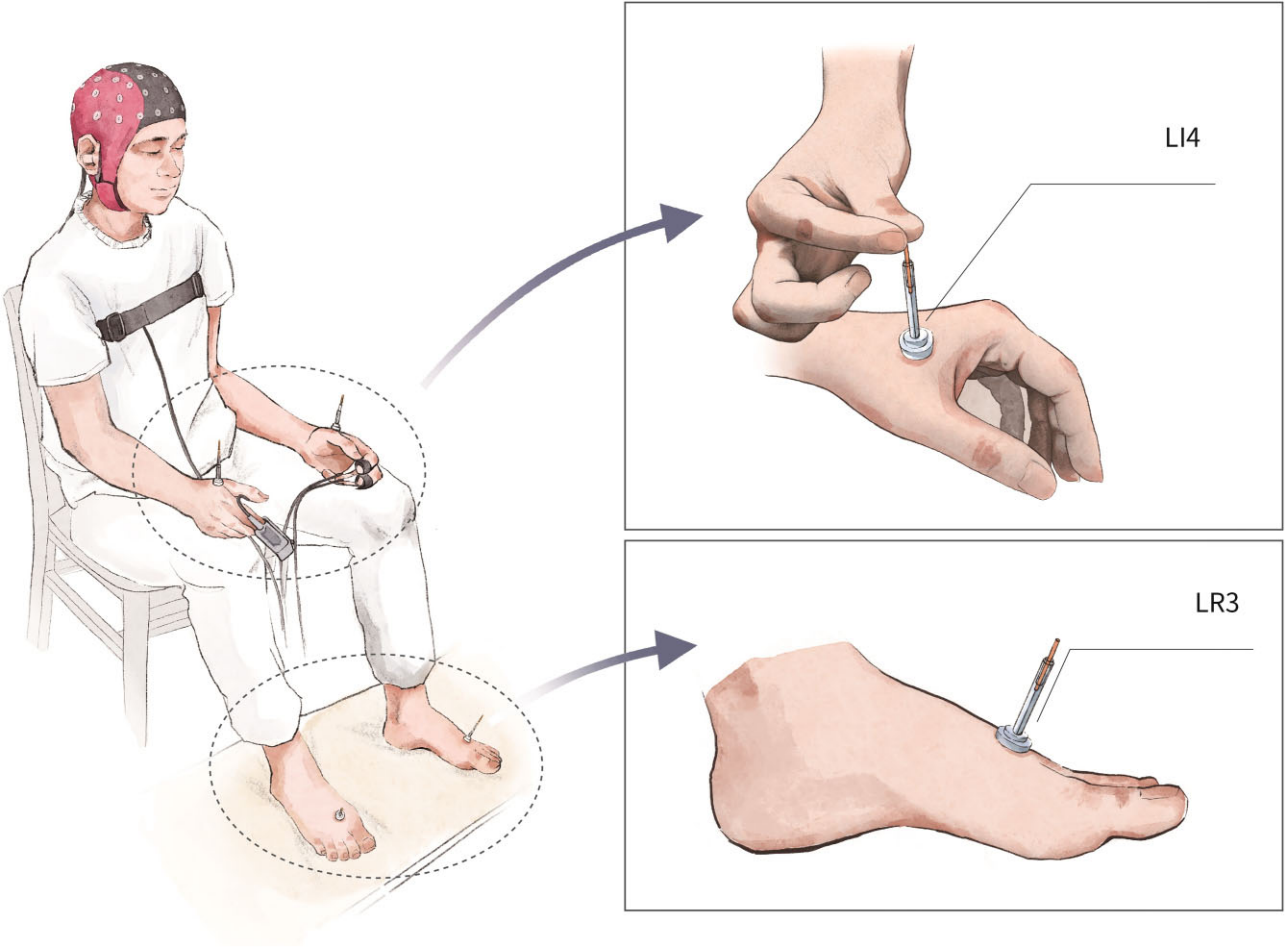
Operation diagram of acupuncture intervention.

One licensed individual delivered acupuncture. He had more than 5 years of clinical experience and attended training before recruitment. Participants received either verum or sham acupuncture with their eyes closed. As illustrated in Fig. 3, a device resembling a plastic tube, similar to the Park device (Park *et al*., 1999), was used to ensure blinding during the procedure. In the verum group, acupuncture was sequentially administered at the right LR3 (Taichong), left LR3, right LI4 (Hegu), and left LI4 acupoints (Fig. 3), which have been shown to alleviate depression symptoms in clinical trials (Li *et al*., 2018; Wen *et al*., 2018; Wu *et al*., 2024). For each point, a single-use sterile Huatuo acupuncture needle (pure silver, 0.25 mm diameter, 40 mm length) was inserted to a depth of 1.5–2 cm and rotated bidirectionally to ~180° at 60 Hz for 1 min. Participants then rested for 10 min with the four needles in place before the acupuncturist removed them. The sham group underwent a similar procedure, but blunt-tipped needles were used without skin penetration. At each acupoint, a single tap on the blunt needle's tail was followed by a 1-min wait with no further manipulation.

### EEG recording and analysis of event-related potentials

Brain electrical activity was recorded using a 64-channel amplifier (eego mylab, ANT Neuro, Berlin, Germany) with CPz as the reference and a sampling frequency of 1000 Hz. Electrode impedances were maintained below 10 kΩ, and no online filters were applied. M1 and M2 (mastoids) channels were removed offline. The EEG data were filtered (0.1 Hz high-pass, 50 Hz low-pass, 48–52 Hz notch), segmented into 3000 ms epochs, and baseline-corrected against the mean voltage over the 1000 ms preceding picture onset. Manual removal and spherical interpolation were used for bad channels, and noisy epochs were manually excluded. Ocular, muscle, and heart artifacts were removed using independent component analysis (ICA) (Delorme *et al*., 2007). Epochs exceeding ±100 µV were automatically rejected. The cleaned EEG data were then re-referenced to the common average.

Event-related potential (ERP) waveforms were time-locked to picture onset, spanning 1200 ms, with a 200 ms pre-stimulus baseline. N2 and P3 components were extracted using principal component analysis (PCA) (Smith *et al*., 2003). The principal components (PCs) and time windows for N2 and P3 were determined manually, based on the PCs’ loading (time distribution), weight (scalp topographic distribution), and prior reports on N2 and P3 (Lou *et al*., 2016; Yuan *et al*., 2009, 2012, 2021). PCA was performed with promax rotation, and the top five PCs, explaining 95.07% of the variance, were extracted. The remaining PCs, with small loadings, were considered noise. The extracted PCs were then back-projected to scalp electrodes (by multiplying component loading by component weight). Frontal (averaged Fz, F1, and F2 electrodes) N1 (80–150 ms), N2 (200–300 ms), and occipital (averaged PO3, PO4, PO5, PO6, PO7, PO8, O1, O2 electrodes) P1 (80–130 ms) and N170 (150–190 ms) were measured with PC3 (explained variance = 3.02%) and PC5 (explained variance = 0.92%) combined. Parietal (averaged Pz, P1, and P2 electrodes) P3 (400–600 ms) was measured with PC1 (explained variance = 79.03%) and PC3 combined.

### Statistical analysis

Data analysis was performed using JASP 0.19.0 and R 4.2.1. Due to statistically significant age differences between groups, analyses were adjusted by including the deviation of age (i.e. age minus grand mean age) as a covariate (Delaney *et al*., 1981). The MASS Index was analyzed using analysis of covariance (ANCOVA). Behavioral, PANAS, and ERP data were subjected to three-way repeated-measures ANCOVA, with degrees of freedom adjusted by the Greenhouse–Geisser correction where necessary. Estimated marginal means (EMMs) with 95% confidence intervals were calculated, and simple effect analysis was performed for significant interactions, with the Bonferroni method applied for multiple test correction. The correlation between changes in PANAS and changes in ERP components was also analyzed.

## Results

### Acupuncture sensation (MASS Index)

**Figure 4:**
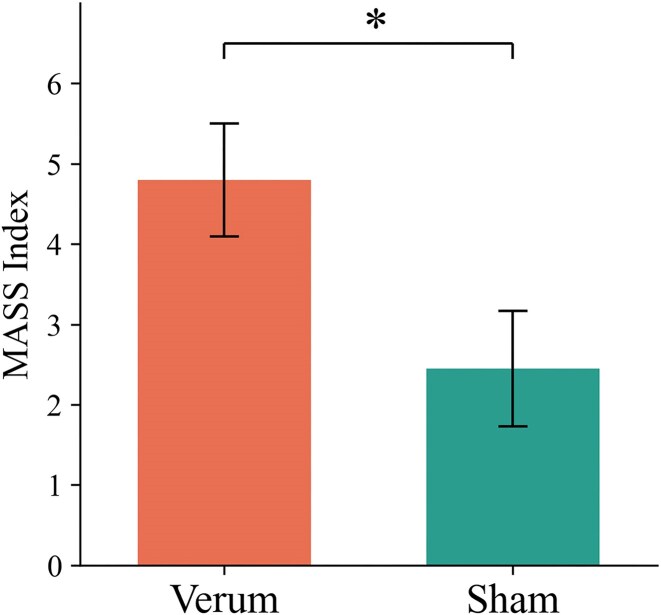
MASS Index of each group. The error bars represent represent the 95% confidence interval.

Verum acupuncture [4.8 (4.1, 5.5)] induced a significantly stronger acupuncture sensation compared to sham acupuncture [2.5 (1.7, 3.2)] [*F*(1, 56) = 23.78, ${\mathrm{\eta }}_p^2$ = 0.274, *P* < 0.001]. The MASS Index for each group is shown in Fig. 4.

### PANAS

Due to a technical issue in web spreadsheet submission, two PANAS questionnaires from the verum group were lost—one from the pre-stimulus report and the other from a separate subject's post-stimulus report. Therefore, a total of 57 questionnaires (27 from the verum group and 30 from the sham group) were included in the following analysis.

**Figure 5:**
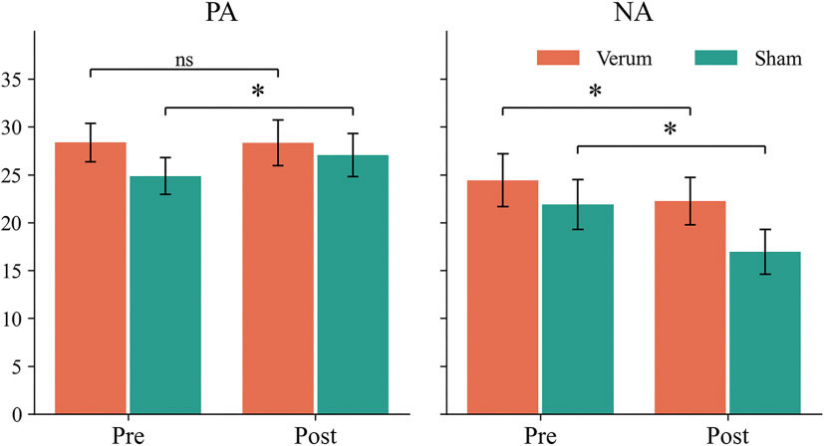
PA (positive effect) and NA (negative effect) scale of each group in the pre- and post-session. Error bars represent the 95% confidence interval.

The main effects of session [*F*(1, 54) = 10.71, ${\mathrm{\eta }}_p^2$ = 0.166, *P* = 0.002], PANA [*F*(1, 54) = 29.59, ${\mathrm{\eta }}_p^2$ = 0.354, *P* < 0.001], and group [*F*(1, 54) = 7.62, ${\mathrm{\eta }}_p^2$ = 0.124, *P* = 0.008], along with the two-way interaction of session × PANA [*F*(1, 54) = 31.54, ${\mathrm{\eta }}_p^2$ = 0.369, *P* < 0.001] and the three-way interaction [*F*(1, 54) = 8.32, ${\mathrm{\eta }}_p^2$ = 0.134, *P* = 0.006], were significant. For negative effect (NA), simple effects showed that group [*F*(1, 54) = 5.30, *P* = 0.025, ${\mathrm{\eta }}_p^2$ = 0.089], session [*F*(1, 54) = 29.73, ${\mathrm{\eta }}_p^2$ = 0.355, *P* < 0.001], and the interaction of group × session [*F*(1, 54) = 4.05, ${\mathrm{\eta }}_p^2$ = 0.065, *P* = 0.049] were significant. The sham group [Estimate = 4.9, *t*(54) = 5.373, Cohen's *d* = 0.680, *P* < 0.001] showed a larger session difference (pre- vs. post-session) than the verum group [Estimate = 2.2, *t*(54) = 2.252, Cohen's *d* = 0.301, *P* = 0.028]. For positive effect (PA), simple effects showed that session [*F*(1, 54) = 5.98, ${\mathrm{\eta }}_p^2$ = 0.100, *P* = 0.018] and the interaction of group × session [*F*(1, 54) = 5.91, ${\mathrm{\eta }}_p^2$ = 0.155, *P* = 0.002] were significant. The sham group showed a significant session difference (pre- vs. post-session) [Estimate = −2.2, *t*(54) = −3.546, Cohen's *d* = −0.299, *P* < 0.001], but the verum group did not. The PA and NA scores for each group in the pre- and post-session are shown in Fig. 5.

### Oddball responses

No significant differences were found between groups, sessions, or intensities for reaction time [578 (555, 602) ms] and accuracy rate [99.2% (99.0%, 99.4%)].

### Occipital P1

**Figure 6:**
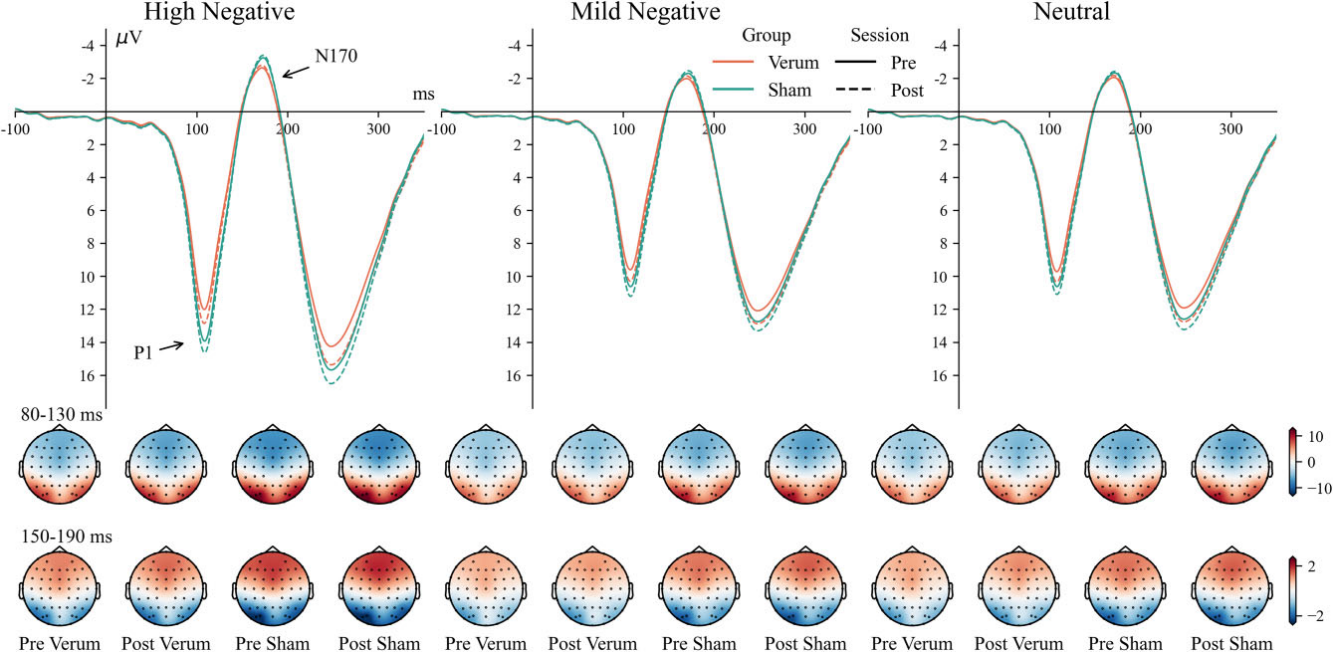
Waveforms and topography of the P1 and N170 components.

The main effect of session was significant [*F*(1, 56) = 8.92, ${\mathrm{\eta }}_p^2$ = 0.137, *P* = 0.004], with larger amplitudes in the post-session [8.1 (7.0, 9.0) µV] compared to the pre-session [7.6 (6.6, 8.7) µV]. The main effect of intensity [*F*(2, 112) = 165.98, ${\mathrm{\eta }}_p^2$ = 0.748, *P* < 0.001] and two way interaction of group × intensity [*F*(2, 112) = 3.27, ${\mathrm{\eta }}_p^2$ = 0.055, *P* = 0.042] were also significant. In the verum group, high-negative pictures [8.2 (6.5, 9.8) µV] evoked larger P1 amplitudes than mild-negative pictures [6.4 (4.8, 7.9) µV, *P *< 0.001] and neutral pictures [6.5 (5.1, 8.0) µV, *P *< 0.001]. In the sham group, high-negative pictures [10.2 (8.6, 11.8) µV] evoked larger P1 amplitudes than mild-negative pictures [8.0 (6.4, 9.5) µV, *P *< 0.001] and neutral pictures [7.9 (6.5, 9.3) µV, *P *< 0.001]. The waveforms and topography of the P1 component are shown in Fig. 6.

### Occipital N170

The main effect of session was significant [*F*(1, 56) = 5.65, ${\mathrm{\eta }}_p^2$ = 0.092, *P* = 0.021], with larger amplitudes in the post-session [−1.7 (−1.9, −1.4) µV] compared to the pre-session [−1.6 (−1.8, −1.3) µV]. The main effect of intensity [*F*(2, 112) = 151.43, ${\mathrm{\eta }}_p^2$ = 0.730, *P* < 0.001] and two-way interaction of group × intensity [*F*(2, 112) = 4.16, ${\mathrm{\eta }}_p^2$ = 0.069, *P* = 0.018] were also significant. In the verum group, high-negative pictures [−1.6 (−2.0, −1.3) µV] evoked larger N170 amplitudes than mild-negative pictures [−1.2 (−1.6, −0.9) µV, *P *< 0.001] and neutral pictures [−1.3 (−1.6, −0.9) µV, *P *< 0.001]. In the sham group, high-negative pictures [−2.2 (−2.6, −1.8) µV] evoked larger N170 amplitudes than mild-negative pictures [−1.7 (−2.0, −1.3) µV, *P *< 0.001] and neutral pictures [−1.7 (−2.0, −1.3) µV, *P *< 0.001]. The waveforms and topography of the N170 component are shown in Fig. 6.

### Frontal N1

**Figure 7:**
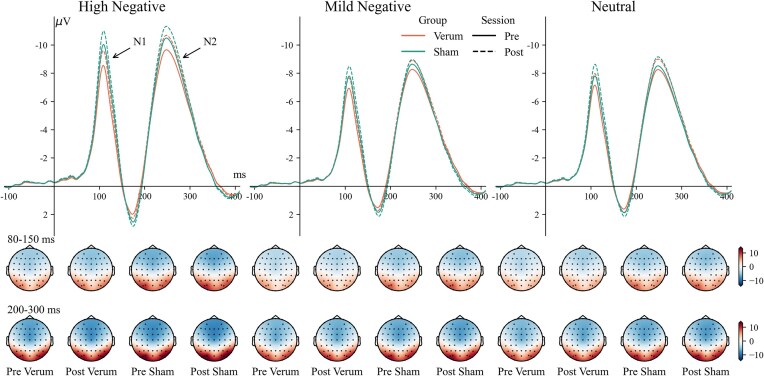
Waveforms and topography of the N1 and N2 components.

The main effect of session was significant [*F*(1, 56) = 19.58, ${\mathrm{\eta }}_p^2$ = 0.259, *P* < 0.001], with larger amplitudes in the post-session [−5.1 (−5.7, −4.5) µV] compared to the pre-session [−4.6 (−5.2, −3.9) µV]. The main effect of intensity [*F*(2, 112) = 121.55, ${\mathrm{\eta }}_p^2$ = 0.685, *P* < 0.001] was also significant. High-negative pictures [−5.6 (−6.3, −4.9) µV] evoked larger N1 amplitudes than mild-negative pictures [−4.4 (−5.0, −3.7) µV, *P *< 0.001] and neutral pictures [−4.5 (−5.1, −3.9) µV, *P *< 0.001]. The waveforms and topography of the N1 component are shown in Fig. 7.

### Frontal N2

The main effect of session was significant [*F*(1, 56) = 18.46, ${\mathrm{\eta }}_p^2$ = 0.248, *P* < 0.001], with larger N2 amplitudes in the post-session [−7.1 (−8.0, −6.0) µV] compared to the pre-session [−6.7 (−7.4, −5.9) µV]. The main effect of intensity was also significant [*F*(2, 112) = 114.58, ${\mathrm{\eta }}_p^2$ = 0.672, *P* < 0.001]. High-negative pictures [−7.7 (−8.5, −6.9) µV] evoked larger N2 amplitudes than mild-negative pictures [−6.5 (−7.3, −5.7) µV, *P *< 0.001] and neutral pictures [−6.5 (−7.2, −5.7) µV, *P *< 0.001]. The waveforms and topography of the N2 component are shown in Fig. 7.

### Parietal P3

**Figure 8:**
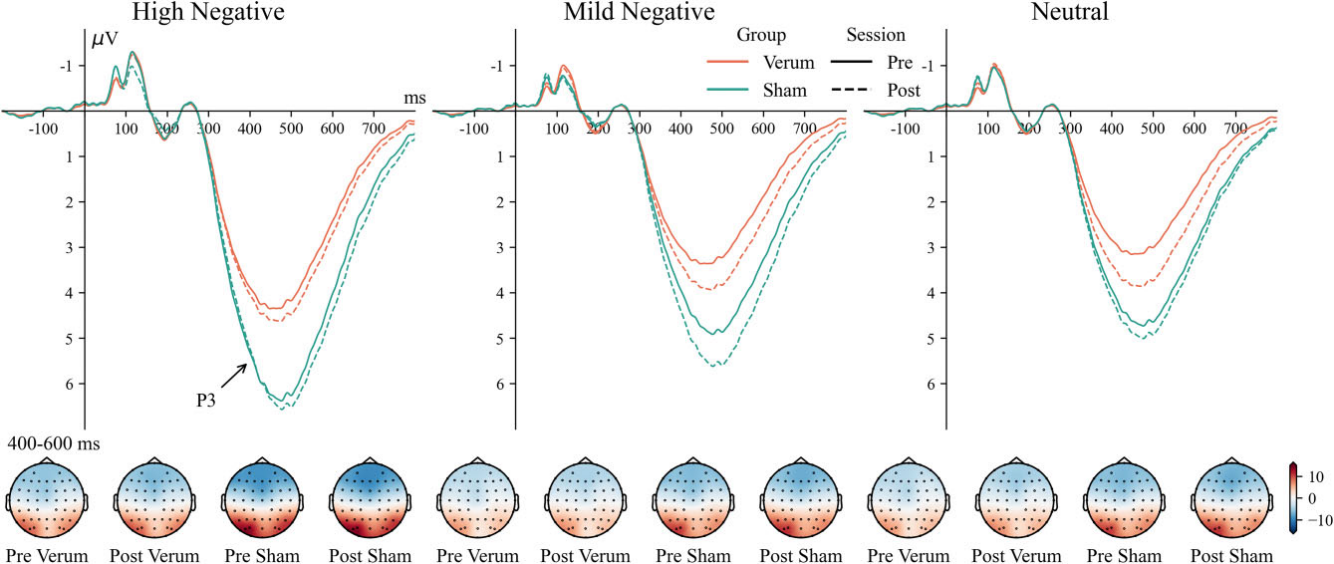
Waveforms and topography of the P3 component.

The main effect of session was significant [*F*(1, 56) = 5.89, ${\mathrm{\eta }}_p^2$ = 0.095, *P* = 0.018], with larger P3 amplitudes in the post-session [4.2 (3.5, 5.0) µV] compared to the pre-session [3.8 (3.0, 4.6) µV]. The main effects of group [*F*(1, 56) = 6.26, *P* = 0.015, ${\mathrm{\eta }}_p^2$ = 0.100] and intensity [*F*(2, 112) = 51.32, ${\mathrm{\eta }}_p^2$ = 0.478, *P* < 0.001], as well as the two-way interaction of group × intensity, were significant [*F*(2, 114) = 6.37, ${\mathrm{\eta }}_p^2$ = 0.102, *P* = 0.001]. In the verum group, high-negative pictures [3.8 (2.1, 5.5) µV] evoked larger P3 amplitudes than mild-negative pictures [3.2 (1.7, 4.7) µV, *P *= 0.088] and neutral pictures [3.2 (1.7, 4.6) µV, *P *= 0.035]. In the sham group, high-negative pictures [7.2 (5.6, 8.9) µV] evoked larger P3 amplitudes than mild-negative pictures [6.1 (4.5, 7.6) µV, *P *< 0.001] and neutral pictures [5.3 (3.9, 6.8) µV, *P *< 0.001], and mild-negative pictures evoked larger P3 amplitudes larger than neutral pictures [*P *= 0.015]. The waveforms and topography of the P3 component are shown in Fig. 8.

### Relationship between PANAS and ERP

In the sham group, PA changes were significantly negatively correlated with occipital N170 (in response to high-negative pictures) changes (*r* = −0.39, *P* = 0.031) and positively correlated with occipital P1 (in response to high-negative pictures) changes (*r* = 0.39, *P* = 0.035), indicating that the more occipital N170 and P1 amplitudes increased, the more PA increased.

## Discussion

In this study, we employed a visual oddball task to simulate naturally occurring threatening emotional events to measure the changes in emotional reactivity before and after receiving acupuncture in young individuals with self-reported depressive symptoms. We found that acupuncture plays a key role in regulating emotional reactivity. Specifically, in ERP-based experiments, occipital P1 and N170, frontal N1, N2, and parietal P3 captured emotional effects, which were primarily represented by increased amplitudes in response to high-negative stimuli compared to neutral stimuli. The amplitudes of all these early- to late-stage components were increased after acupuncture, indicating a broad regulatory effect involving multiple processing stages. Surprisingly, acupuncture did not regulate the response to negative stimuli alone; instead, it regulated the response to both negative and neutral stimuli equally, indicating a generalized regulatory effect. Interestingly, and contrary to our initial expectations, sham acupuncture also exerted a substantial regulatory effect similar to verum acupuncture, suggesting these effects may not depend solely on needle penetration.

Frontocentral N2 activity observed in the oddball paradigm is commonly interpreted as a neural marker of oriented attention, particularly sensitive to unexpected and potentially harmful stimuli (Bahm *et al*., 2017; Carretié *et al*., 2004; Clayson *et al*., 2012; Kypriotakis *et al*., 2020; Lou *et al*., 2016; Nagy *et al*., 2003). The P3 component reflects cognitive evaluation processes involved in assessing the significance of emotional information (Ito *et al*., 1998; Yuan *et al*., 2007, 2009). Continuous negative emotions disrupt the ability of individuals with depression to recruit cognitive resources effectively, leading to impairments in attentional systems (Holmes and Pizzagalli, 2008; Quinn *et al*., 2018; Rock *et al*., 2014; Shao *et al*., 2020; Snyder, 2013; Vilgis *et al*., 2015) and cognitive resource allocation (Elliott *et al*., 2002; Gao *et al*., 2022; Joormann and Stanton, 2016; Starr *et al*., 2020). This impairment is reflected in reduced N2 and P3 amplitudes (Chen and Li, 2023; Dai and Feng, 2011; Magee *et al*., 2023; el Massioui and Lesèvre, 1988; Nan *et al*., 2018; Tang *et al*., 2011; Thomas *et al*., 2007), indicating diminished attention and reduced evaluative processing of emotional stimuli. The current findings suggest that acupuncture may help restore these cognitive processing mechanisms, thereby regulating emotional responses.

The occipital P1 and its frontal counterpart (frontal N1) peak ~100 ms after stimulation, sensitive to the emotional and physical properties of the stimuli and influenced by attention. In addition, temporal occipital N170 is known to be sensitive to emotional faces and also indexes emotional processing of complex situational pictures (Schettino *et al*., 2016). These components are involved in early visual processing of emotional stimuli. A recent study (Wang Q *et al*., 2022) showed that adolescents with self-reported depression responded to picture stimuli with significantly lower N1 (120–190 ms) component amplitude than healthy individuals. Importantly, they found no significant interactions, suggesting that across a wide range of regions (frontal, central, parietal), for different stimulus types (positive, dysphoric, threatening, and neutral), a generalized decrease in N1 activity occurred. This suggests that people with depression allocate fewer attention resources to incoming visual stimuli regardless of emotional valence. The decrease of emotional reactivity in depressed people may result from reduced generalized visual processing. This finding is supported by functional magnetic resonance imaging studies showing that visual cortex function is diminished in MDD patients (Li *et al*., 2013; Peng *et al*., 2011). Depressive disorders may therefore involve not only emotional symptoms but also fundamental changes in information processing. The results of the current study suggest an effective means to modulate the visual processing of pictures in young people with depressive symptoms.

The blunted emotional reactivity in depression reflects a general disengagement from the environment (Bylsma *et al*., 2021). After the intervention, the general increase in the amplitude of ERP components from early- to late-stage processing, in response to both negative and neutral stimuli, suggests a generalized modulatory effect. We suspect that this effect may stem from the regulation of cortical excitability, attention, and arousal, thereby restoring an individual's response to external stimuli. For example, acupuncture at LR3 or other vision and non-vision-related acupoints strongly activates the visual cortex (Liu *et al*., 2012; Zhang *et al*., 2009). Moreover, acupuncture and pain stimuli can enhance both alerting and executive network efficiency in attention networks of healthy individuals (Liu *et al*., 2013). Thus, acupuncture-induced improvements in emotional reactivity may result from modulation of the frontoparietal attention network, enhancing attention towards external stimuli and their evaluative processing. Additionally, acupuncture has demonstrated rapid antidepressant effects by inhibiting M1-ACh receptors on prefrontal cortex (PFC) GABAergic interneurons, regulating glutamate levels and triggering downstream neural mechanisms (Ning *et al*., 2024). This regulation of the PFC GABA/glutamate balance enhances the functional stability of the ventral tegmental area (VTA) – nucleus accumbens (NAc) – medial prefrontal cortex (mPFC) network (Li *et al*., 2022), thereby further modulating motivation-based arousal. Further research is warranted to empirically validate these potential mechanisms.

Deqi, traditionally considered a crucial aspect of acupuncture's effectiveness (Kong *et al*., 2007), has garnered significant interest in distinguishing between verum and sham acupuncture effects. In our study, the verum acupuncture group reported significantly stronger Deqi sensations than the sham group, suggesting a more noticeable sensory experience (Kong *et al*., 2007). Interestingly, despite these differences in sensory perception, the ERP component results were remarkably similar in both groups. Previous studies have explored the similarities in stimulus effects between verum and sham acupuncture. In clinical acupuncture practice, the sensory discrimination and affective dimensions of social touch are considered important for treatment outcomes (Chae and Olausson, 2017). The therapeutic effects of sham needles, involving enhanced tactile stimulation and direct activation of somatosensory pathways, have been extensively documented (Chae *et al*., 2018). Despite the reduced somatosensory input of sham acupuncture compared to verum acupuncture, the cognitive aspects of sham procedures may closely resemble those of actual acupuncture treatments. This similarity could explain the comparable efficacy observed between sham and verum acupuncture (Lee and Chae, 2021). Thus, the similar outcomes in our investigation may arise from the activation of shared neural circuits involved in cognitive processing and emotional management. The therapeutic effects of acupuncture, whether verum or sham, may partly stem from a generalized modulation of the central nervous system, involving both specific and non-specific effects, rather than solely from the mechanical effects of needle insertion. The results of the current study suggest that although participants reported different subjective sensations, the impact of acupuncture on functions in the brain may be similar, highlighting a more complex interaction of factors underlying acupuncture's therapeutic advantages. This highlights the necessity in future acupuncture research of systematically incorporating psychological frameworks and assessment measures to elucidate the complex “mind–body–context” interaction, which is a key focus of the “acupuncture psychology.” For instance, it may be promising to amplify depressive intervention effects by combining acupuncture intervention with other updated psychotherapies, such as automatic emotion regulation (Li *et al*., 2024).

Changes in self-reported emotions were observed after the intervention in both groups. Positive emotions were maintained (verum) or increased (sham), while negative emotions decreased (with a greater reduction in the sham group than the verum group), indicating an overall improvement in emotional state after intervention. Unexpectedly, sham acupuncture led to better emotional rehabilitation. To simulate the experience of verum acupuncture, sham acupuncture stimulates surface touch receptors and activates C-tactile fibers (Chae *et al*., 2018). Notably, both penetrating and non-penetrating skin stimulation can result in self-reported feelings of pleasure, an effect linked to C-tactile touch (Field, 2019; Lee *et al*., 2023; Takakura *et al*., 2013), suggesting that surface stimulation with different forms or parameters may activate C-tactile fibers and induce related emotional effects. In fact, sham acupuncture has been widely reported to produce mood rehabilitation effects similar to those of real acupuncture (Chang *et al*., 2023; Deng *et al*., 2024; Wang L *et al*., 2022; Zhao *et al*., 2023), and in some cases it may even produce better effects than verum acupuncture (Zucker *et al*., 2017). We suspect that the mood improvement observed in this study may result from the combined effect of the entire acupuncture procedure, influenced by various factors in both verum and sham treatments. For instance, verum acupuncture may induce stronger anxiety than sham acupuncture (Yoo *et al*., 2007), which could partially offset its mood-improving effects. The current findings do not deny the efficacy of acupuncture but instead emphasize the importance of non-specific effects of acupuncture. In particular, intensity dependence of acupuncture treatment has been well established (Liu *et al*., 2020), and acupuncture should be performed with appropriate stimulation intensity based on actual clinical conditions. The results of the current study may suggest that an appropriate, mild Deqi sensation could induce better emotional regulation, and in the context of emotional blunting, this regulation may be related to the enhancement of visual processing.

Attention was drawn to the observed increase in emotional reactivity to negative stimuli (measured by ERP), alongside a decrease in NA in PANAS scores after the intervention. In this study, participants completed a non-emotional task (distinguishing between standard and deviant stimuli) that implicitly presented emotional stimuli (in the deviant condition). Participants were not specifically instructed to complete the PANAS questionnaire in relation to the negative stimuli of the oddball paradigm; instead, they were asked to respond according to their current mood state. In fact, the oddball task and the PANAS assessment might have been completed in different contexts. Furthermore, ERP components reflect neurobiological responses, while PANAS scores reflect subjective experience and appraisal. These two aspects denote different facets of emotion that do not necessarily correlate. It has become increasingly clear that facets of emotion across different response domains are only loosely coupled (Bylsma *et al*., 2016; Bylsma, 2021; Mauss *et al*., 2005). Physiological changes can occur without corresponding experiential changes, and vice versa (Kreibig, 2010; Yuan *et al*., 2015). Therefore, the increase in neural responses to negative emotional stimuli after the intervention may not be inconsistent with the decrease in self-reported negative emotion. There is another possible explanation that self-reported improvement in emotion may result from breaking away from emotional blunting.

## Conclusion

Emotional reactivity in youth with depressive symptoms was altered by acupuncture. Although this effect may be non-specific, it still highlights the important role of acupuncture in the prevention and treatment of depression in youth.

## Data Availability

The data generated during and/or analyzed during the current study are available from the corresponding author on reasonable request.
